# Longitudinal functional and imaging outcome measures in FKRP limb-girdle muscular dystrophy

**DOI:** 10.1186/s12883-020-01774-5

**Published:** 2020-05-19

**Authors:** Doris G. Leung, Alex E. Bocchieri, Shivani Ahlawat, Michael A. Jacobs, Vishwa S. Parekh, Vladimir Braverman, Katherine Summerton, Jennifer Mansour, Genila Bibat, Carl Morris, Shannon Marraffino, Kathryn R. Wagner

**Affiliations:** 1 Center for Genetic Muscle Disorders, Hugo W. Moser Research Institute at Kennedy Krieger Institute, 716 North Broadway, Room 411, Baltimore, MD 21205 USA; 2 Department of Neurology, Johns Hopkins University School of Medicine, Baltimore, MD USA; 3 Department of Computer Science, Johns Hopkins University, Baltimore, MD USA; 4 Department of Radiology and Radiological Science, Johns Hopkins University School of Medicine, Baltimore, MD USA; 5 The Sidney Kimmel Comprehensive Cancer Center, Johns Hopkins University School of Medicine, Baltimore, MD USA; 6 Tulane University School of Medicine, New Orleans, LA USA; 7 Solid Biosciences, Cambridge, MA USA; 8 Pfizer, Inc., Cambridge, MA USA; 9 Department of Neuroscience, Johns Hopkins University School of Medicine, Baltimore, MD USA

**Keywords:** FKRP, Whole-body MRI, Limb-girdle muscular dystrophy, Biomarkers, Deep learning, Convolutional neural network, Tissue signatures

## Abstract

**Background:**

Pathogenic variants in the FKRP gene cause impaired glycosylation of α-dystroglycan in muscle, producing a limb-girdle muscular dystrophy with cardiomyopathy. Despite advances in understanding the pathophysiology of FKRP-associated myopathies, clinical research in the limb-girdle muscular dystrophies has been limited by the lack of normative biomarker data to gauge disease progression.

**Methods:**

Participants in a phase 2 clinical trial were evaluated over a 4-month, untreated lead-in period to evaluate repeatability and to obtain normative data for timed function tests, strength tests, pulmonary function, and body composition using DEXA and whole-body MRI. Novel deep learning algorithms were used to analyze MRI scans and quantify muscle, fat, and intramuscular fat infiltration in the thighs. T-tests and signed rank tests were used to assess changes in these outcome measures.

**Results:**

Nineteen participants were observed during the lead-in period for this trial. No significant changes were noted in the strength, pulmonary function, or body composition outcome measures over the 4-month observation period. One timed function measure, the 4-stair climb, showed a statistically significant difference over the observation period. Quantitative estimates of muscle, fat, and intramuscular fat infiltration from whole-body MRI corresponded significantly with DEXA estimates of body composition, strength, and timed function measures.

**Conclusions:**

We describe normative data and repeatability performance for multiple physical function measures in an adult FKRP muscular dystrophy population. Our analysis indicates that deep learning algorithms can be used to quantify healthy and dystrophic muscle seen on whole-body imaging.

**Trial registration:**

This study was retrospectively registered in clinicaltrials.gov (NCT02841267) on July 22, 2016 and data supporting this study has been submitted to this registry.

## Background

The limb-girdle muscular dystrophies are a class of genetic muscle diseases that are characterized by progressive muscle weakness. Several limb-girdle muscular dystrophies are caused by pathogenic variants in genes that regulate glycosylation of α-dystroglycan in the sarcolemma. Fukutin-related protein (FKRP) is a glycosyltransferase that is necessary for linkage of membrane-bound proteins to the basal lamina [1]. Pathogenic variants in FKRP cause an autosomal recessive muscular dystrophy that is most commonly known as limb-girdle muscular dystrophy 2I (LGMD2I), although emerging classification schemes also refer to this disease as LGMD R9 [2]. LGMD2I is a rare disease; however, it is one of the more common forms of muscular dystrophy in Northern Europe owing to a prevalent founder variant (NM_024301.5(FKRP): c.826C > A (p.Leu276Ile)) [3, 4]. There is currently no curative medical treatment for LGMD2I, and the management is primarily supportive.

There is wide variability in the clinical severity of LGMD2I, and it is uncertain whether the functional outcome measures that have been used in trials for other neuromuscular diseases can be applied to trials of LGMD2I. To characterize these outcome measures in the LGMD2I population, we observed a group of adults with LGMD2I during an untreated 4-month lead-in period to a phase 1b/2a clinical trial. This lead-in period was incorporated into the trial protocol to allow investigators to evaluate the repeatability of several commonly used neuromuscular outcome measures and to determine if changes could be detected in these outcome measures over a brief period of observation.

To evaluate an exploratory outcome measure, trial participants were also studied using a whole-body magnetic resonance imaging (WBMRI) protocol which included chemical shift (Dixon) sequences [5]. The images from these sequences were analyzed using a multiparametric deep learning (MPDL) algorithm designed to identify unique tissue signatures for various organ types [6–8]. Deep learning algorithms are increasingly being used for segmentation and classification of radiological images [9, 10]. Using the MPDL algorithm, we were able to derive estimates of muscle, fat, and intramuscular fat infiltration. We examined the associations between these MRI measurements and clinical outcome measures.

## Methods

### Study design and setting

This observational cohort study was a part of a clinical trial protocol (NCT02841267) that was approved by the Institutional Review Board at the Johns Hopkins University School of Medicine. Data collection took place at the Kennedy Krieger Institute in Baltimore, Maryland (an affiliate of the Johns Hopkins Medical Institutions). All participants were screened and enrolled in the study between July 15, 2016 and April 13, 2017. Adults ages 18 and older with LGMD2I confirmed by genetic testing were invited to participate. Because participants were expected to proceed from the lead-in period into a treatment period with a non-FDA approved drug, the eligibility criteria excluded people who were unable to walk 10 m or rise from a chair independently, had hepatic or renal impairment, were pregnant or nursing, had cognitive or psychiatric limitations that prevented informed consent or compliance with study procedures, had received corticosteroids or other investigational therapies, had a left ventricular ejection fraction < 50% by echocardiogram, had a history of coagulation disorder or other contraindication to muscle biopsy, or had contraindications to MRI scanning.

### Demographic data and clinical measurements

At the time of enrollment, age, sex, race/ethnicity, age of symptom onset, height, weight, genotype, and creatine kinase (CK) level were recorded. A licensed physical therapist performed the following physical function tests: manual muscle testing (MMT) using the Medical Research Council (MRC) muscle scoring system, quantitative muscle testing (QMT) using a handheld dynamometer (Microfet), two-minute walk distance (2MWD), ten meter walk/run (10MWR), timed up-and-go (TUG), four stair climb (4SC), and the Performance of Upper Limb (PUL) assessment [11]. A two-minute walk distance was selected over a six-minute walk test because it was anticipated that a number of eligible participants would be unable to walk continuously for 6 minutes. Prior studies have indicated that performance on the two-minute walk test strongly correlates with the six-minute walk distance [12, 13]. Twenty-two muscle groups were tested using the MMT (neck flexors, neck extensors, and bilateral shoulder abductors, elbow flexors, elbow extensors, hip flexors, hip extensors, hip abductors, knee extensors, knee flexors, ankle dorsiflexors, ankle plantar flexors), and each muscle group was assigned a score of 1 through 12 based on an adapted form of the MRC scoring system [14, 15]. The scores from all 22 muscle groups were added to arrive at a total MMT score (with a maximum possible score of 264). Quantitative muscle testing was performed to determine strength in the following muscle groups: shoulder abductors, elbow flexors, elbow extensors, hip abductors, and knee extensors; these values were not combined for analysis. Pulmonary function testing using a bedside spirometer was performed by the clinical evaluator and forced vital capacity (FVC), forced expiratory volume (FEV1), maximal inspiratory pressure (MIP), and maximal expiratory pressure (MEP) measurements were obtained. Three trials of pulmonary function testing were performed at each visit, and the best of the three trials was used for analysis. A dual energy x-ray absorptiometry (DEXA) scan was performed to obtain lean body mass and body fat measurements over the whole body.

The clinical measurements were obtained at baseline and repeated at a follow-up visit 4 months later. Each follow-up visit took place over two consecutive days, and the 2MWD, 10MWR, TUG, 4SC, and PUL were performed on both days to allow for repeatability analysis [16–19].

### Magnetic resonance imaging acquisition

Whole-body MRI scans were acquired using a 3 Tesla Prisma scanner (Siemens, Erlangen, Germany) with continuous table movement (CTM) capabilities [20]. Participants were scanned head first and in the supine position. Three radiofrequency coils were used: a peripheral angiography matrix coil that was placed over the legs and two body matrix coils that were placed over the chest and abdomen/pelvis. Images of the chest and abdomen were acquired over a series of 16-s breath holds to minimize motion artifact from the chest wall and diaphragm. Dixon images were acquired as part of a longer protocol that included T_1_-weighted, short-tau inversion recovery (STIR), and diffusion-weighted sequences that were not quantitatively analyzed. Images were acquired using CTM sequences that scanned a total length of 1170 mm starting from the shoulder. Dixon scan parameters were: TR/TE = 200/3.69 ms and 4.92 ms for opposed- and in-phases, flip angle = 70°, FOV = 500x500mm, slice thickness = 5 mm.

### Deep learning and image post-processing

#### Multiparametric deep learning tissue signatures

The MPDL network builds a composite feature representation using the muscular dystrophy tissue signatures defined by a tissue signature vector (TS) as gray level intensity values corresponding to each voxel position within the images and defined by the equation below:

$$ MPDL\ Tissue\ Signature=T{S}^{\left(\tau \right)}={\left[{In\ phase}_n^{\left(\tau \right)}, Out\ {of\ phase}_n^{\left(\tau \right)},{Water}_n^{\left(\tau \right)},{Fat}_n^{\left(\tau \right)}\right]}^T $$

Here, *(ι)* is the tissue type, *n* is the number of the images in the sequence, and T = transpose.

**Fig. 1:**
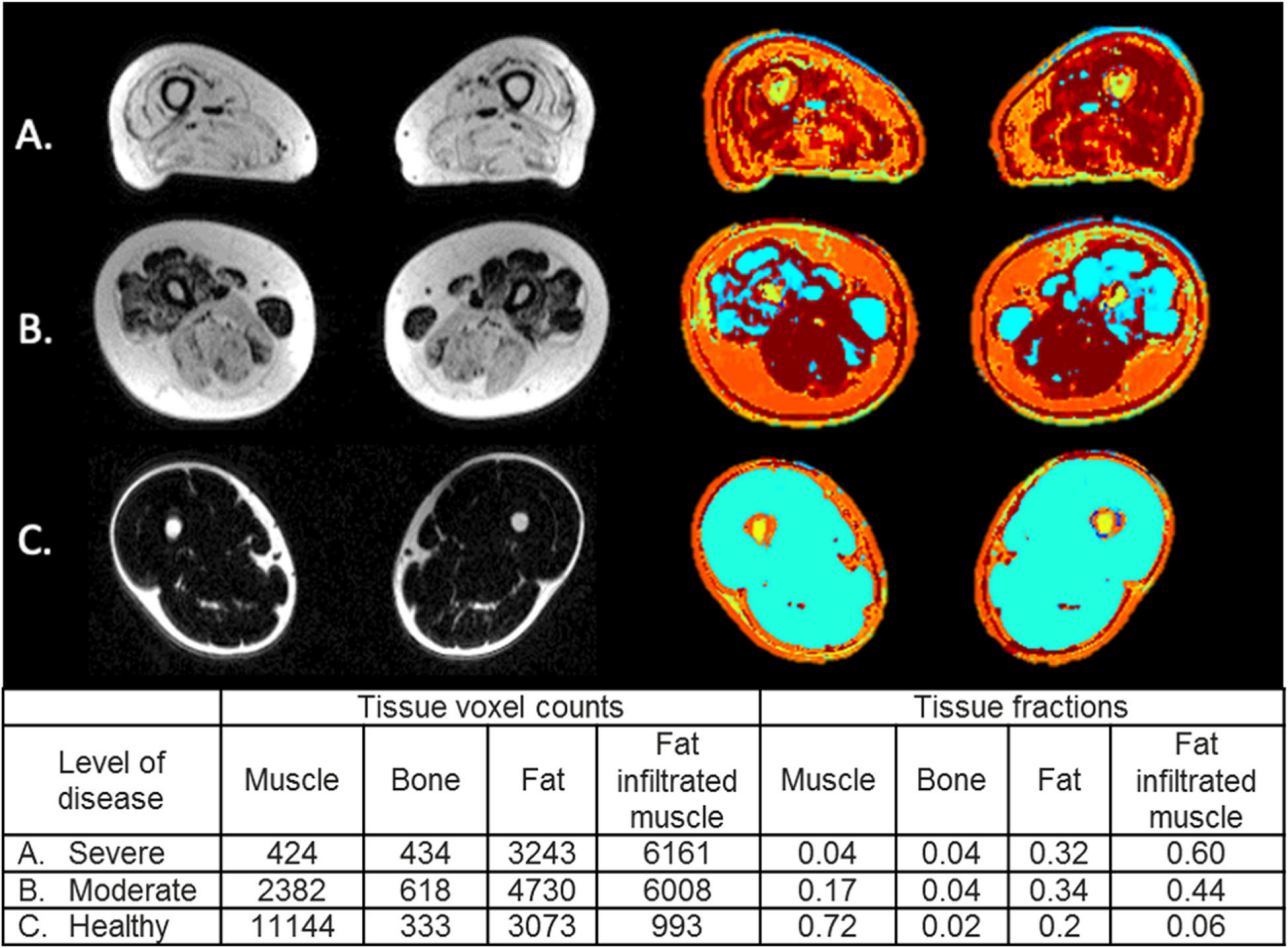
Demonstration of the MPDL tissue signature model and CNN segmentation mapping in participants with (**a**) severe fat infiltration and (**b**) moderate fat infiltration at the level of the mid-thigh. An image of a healthy volunteer (**c**) is provided for comparison. The color scale is coded as follows: healthy muscle is blue, bone is yellow, fat is orange, and fat infiltrated muscle is red. The number of voxels and the fraction of total voxels corresponding to each tissue type are shown in the table

The TS vector models the inter-parametric relationships and the intensity values of each of the tissue types in the MR images. These TS are used to segment different tissue types such as bone, muscle, fat, and fat infiltrated muscle. The MPDL network was trained on both normal and muscular dystrophy tissue signatures derived from MR images that were not collected as part of the study (Fig. 1) [21–23]. The TS are used as inputs into the deep learning algorithm to define each tissue type and enable the deep learning model to define tissue signatures of subcutaneous fat and fat infiltrated muscle.

#### Convolutional neural network

The patch-based convolutional neural network (CNN) was implemented and trained on the TS image patches of size 5 × 5xN corresponding to each N dimensional tissue signature of different tissues in the thigh. The 5 × 5 image patch of a tissue signature corresponds to the immediate 5 × 5 neighborhood of that voxel position. The 2D-CNN consisted of four layers with 128, 64, 32 and 16 filters respectively, followed by a fully connected layer and a softmax layer [24]. Inputs to the convolutional layers were zero padded so that the output has the same size as the input. Each convolutional layer had a kernel size of 3 × 3 and was followed by a batch normalization and a ReLU layer. Additionally, max pooling layers with a kernel size of 2 × 2 and a stride of 2 × 2 were applied following the first two convolutional layers. The cross-entropy loss function was used for the error rate analysis. The 2D-CNN was trained for thirty epochs using the adam optimizer with a learning rate of 0.001, momentum = 0.9, β1 = 0.9, β2 = 0.999, ε = 10^− 8^, and minibatch size of 1024 [25].

#### Muscular dystrophy tissue evaluation

We focused our analysis on the subset of images from the WBMRI scans that corresponded to the muscle groups in the thighs, which are the most frequently scanned anatomic regions in imaging studies of skeletal muscle. Using the TS model, tissue signatures for healthy muscle, subcutaneous fat, and fat-infiltrated muscle were defined based on the gray level intensity values of the multiple co-registered images (in phase, out of phase, fat, and water) obtained using the Dixon imaging protocol. Based on the segmentations of the different tissue types, we were able to derive tissue fractions for each of the major tissue types of muscle, fat, fat-infiltrated muscle, and bone. Verification of appropriate tissue segmentation was performed by a board-certified musculoskeletal radiologist (SA) who compared automated segmentation images to co-registered anatomic images via visual inspection. The intramuscular fat and muscle fractions that were used in the longitudinal analysis were calculated by dividing the total number of voxels corresponding to fat infiltrated or healthy muscle by the total number of muscle voxels (both healthy and fat infiltrated). The voxels corresponding to bone and subcutaneous fat were excluded from this calculation. Had they been included, subjects with more subcutaneous fat would appear to have reduced intramuscular fat and muscle fractions compared to subjects with less subcutaneous fat even though the degree of intramuscular fat infiltration could be similar. As the body mass index ranged from 15.0 to 30.3 in this study, the amount of subcutaneous fat was felt to be a significant potential confounder.

### Statistical methods

A target sample size of 20 enrollees was established based on the feasibility of recruitment and appropriateness for a phase 1b/2a safety trial.

Outcome measure data were explored using histograms and scatter plots. Shapiro-Wilk testing was performed to determine if variables met the assumption of normality. Bland-Altman plots were constructed for variables for which repeatability data were available to assess for uniform variance across the data ranges. Spearman correlation coefficients were calculated for the outcomes that underwent repeatability testing. To assess changes in outcome measures over the 4-month lead-in period, paired t-tests were performed when variables met the assumption of normality, and Wilcoxon signed rank tests for matched pairs were performed for variables that did not. Thigh fat fractions were plotted against MMT sum scores, all timed function tests, and pulmonary function tests. The numbers of voxels associated with total body fat and total body muscle were compared to body composition measurements obtained through DEXA scanning. Correlation coefficients were calculated when the associations between outcomes were reasonably linear. Statistical significance was set at *p* < 0.05 with Bonferroni adjustment for multiple comparisons.

## Results

### Participants

Twenty participants were recruited for the study and completed the first visit. One subject withdrew from the study shortly after enrollment and did not complete any subsequent visits and was therefore not included in the longitudinal analysis. Demographic information for the remaining 19 participants is reported in Table 1. All participants identified as Caucasian. Three participants did not complete the physical function evaluation on both days of the follow-up visit and therefore did not contribute to the repeatability analysis of the 2MWD, 10MWR, TUG, 4SC, and PUL. One participant did not complete the WBMRI scan at the initial visit, and one participant did not complete the WBMRI scan at the follow-up visit.

**Table 1:** Summary data for study participants

Participant	Sex	Age (years)	Body mass index	Age at symptom onset	Allele 1 Pathogenic variant	Allele 2 Pathogenic variant	Creatine kinase (U/L)
1	F	38	21.2	29	NM_024301.5(FKRP): c.826C > A (p.Leu276Ile)	NM_024301.5(FKRP): c.826C > A (p.Leu276Ile)	2423
2	F	53	21.0	22	NM_024301.5(FKRP): c.826C > A (p.Leu276Ile)	NM_024301.5(FKRP): c.826C > A (p.Leu276Ile)	488
3	M	51	27.9	38	NM_024301.5(FKRP): c.826C > A (p.Leu276Ile)	NM_024301.5(FKRP): c.826C > A (p.Leu276Ile)	5668
4	F	38	21.1	28	NM_024301.5(FKRP): c.826C > A (p.Leu276Ile)	NM_024301.5(FKRP): c.826C > A (p.Leu276Ile)	951
5	F	65	26.6	35	NM_024301.5(FKRP): c.826C > A (p.Leu276Ile)	NM_024301.5(FKRP):c.1073C > T (p.Pro358Leu)	202
6	F	33	20.3	22	NM_024301.5(FKRP): c.826C > A (p.Leu276Ile)	NM_024301.5(FKRP): c.826C > A (p.Leu276Ile)	1506
7	M	41	16.1	25	NM_024301.5(FKRP): c.826C > A (p.Leu276Ile)	NM_024301.5(FKRP):c.1433 T > C (p.Ile478Thr)	2832
8	F	26	27.0	20	NM_024301.5(FKRP): c.826C > A (p.Leu276Ile)	NM_024301.5(FKRP): c.826C > A (p.Leu276Ile)	4579
9	F	23	15.0	15	NM_024301.5(FKRP): c.826C > A (p.Leu276Ile)	NM_024301.5(FKRP): c.826C > A (p.Leu276Ile)	2399
10	M	30	22.3	26	NM_024301.5(FKRP): c.826C > A (p.Leu276Ile)	NM_024301.5(FKRP): c.826C > A (p.Leu276Ile)	981
11	M	67	24.5	40	NM_024301.5(FKRP): c.826C > A (p.Leu276Ile)	NM_024301.5(FKRP):c.586G > C (p.Gly196Arg)	226
12	F	23	23.0	17	NM_024301.5(FKRP): c.826C > A (p.Leu276Ile)	NM_024301.5(FKRP): c.826C > A (p.Leu276Ile)	1726
13	M	34	21.5	20	NM_024301.5(FKRP): c.826C > A (p.Leu276Ile)	NM_024301.5(FKRP): c.826C > A (p.Leu276Ile)	3983
14	F	41	30.3	21	NM_024301.5(FKRP): c.826C > A (p.Leu276Ile)	NM_024301.5(FKRP): c.826C > A (p.Leu276Ile)	3072
15	F	47	27.1	16	NM_024301.5(FKRP): c.826C > A (p.Leu276Ile)	NM_024301.5(FKRP): c.826C > A (p.Leu276Ile)	222
16	M	32	21.7	14	NM_024301.5(FKRP): c.826C > A (p.Leu276Ile)	NM_024301.5(FKRP):c.1087G > T (p.Val363Leu)	1716
17	F	44	20.2	15	NM_024301.5(FKRP): c.826C > A (p.Leu276Ile)	NM_024301.5(FKRP): c.826C > A (p.Leu276Ile)	1099
18	F	29	24.8	18	NM_024301.5(FKRP): c.826C > A (p.Leu276Ile)	NM_024301.5(FKRP): c.826C > A (p.Leu276Ile)	1428
19	M	19	27.6	5	NM_024301.5(FKRP): c.826C > A (p.Leu276Ile)	NM_024301.5(FKRP): c.826C > A (p.Leu276Ile)	4803

### Analysis of clinical outcomes

**Fig. 2:**
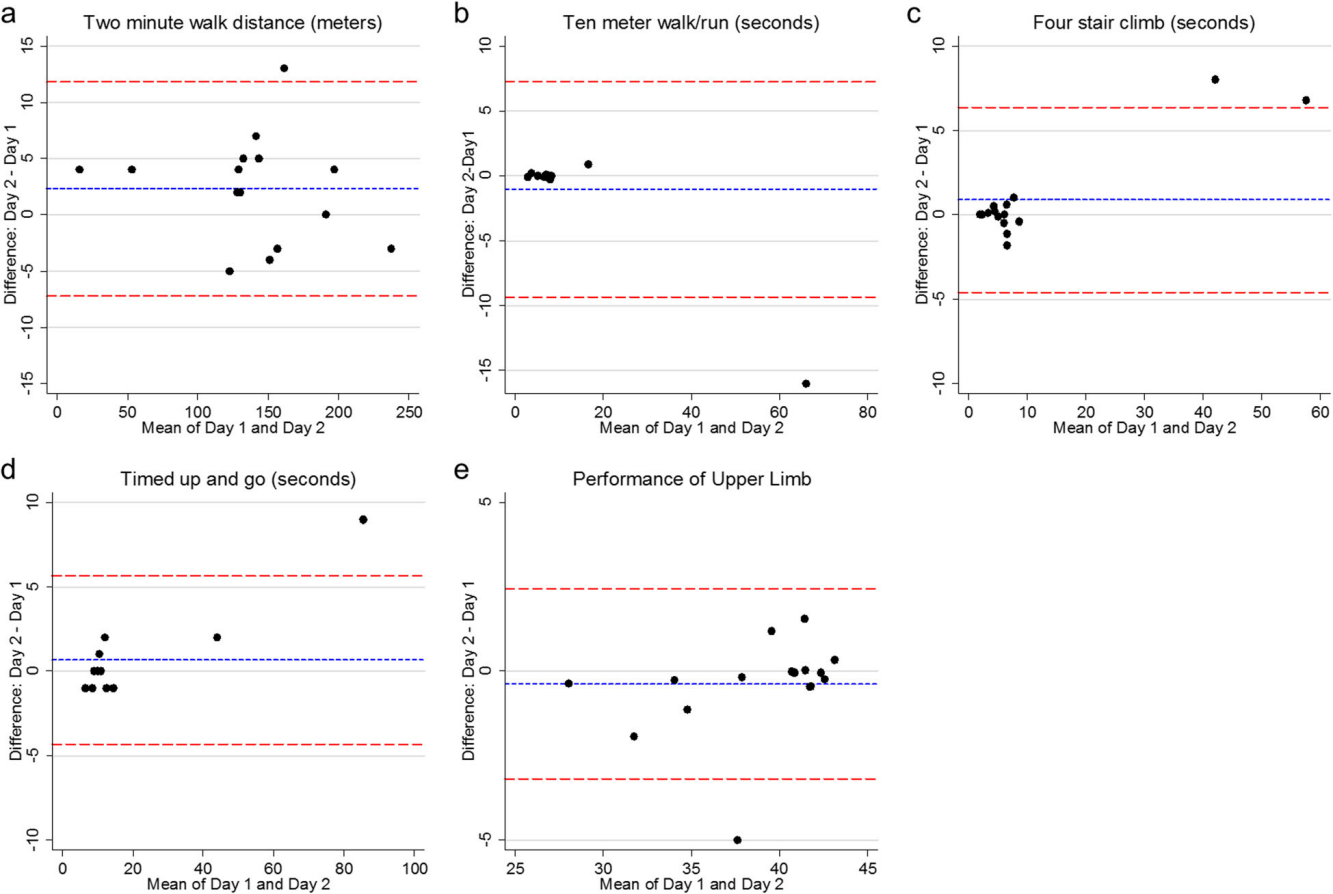
Bland-Altman plots of five timed function tests that underwent repeatability testing on two consecutive days. Blue dashed lines represent the mean of the difference between consecutive tests. Red lines show the 95% limits of agreement for the differences between consecutive tests

Repeatability analysis: 2MWD, 10MWR, TUG, 4SC, PUL tests were performed on each of two consecutive days during the follow-up visit to allow for repeatability testing (Table 2). Spearman correlation coefficients for all five outcome measures were > .90 with *p*-values < 0.0001, suggesting a strong linear association between tests performed on consecutive days. However, Bland-Altman graphs of the TUG, 4SC, and 10MWR also show outlying values at the more severe end of the disease spectrum (Fig. 2).

**Table 2:** Summary statistics for baseline, follow-up, and repeatability measurements

	Baseline			16- week follow-up (Day 1)					16-week follow-up (Day 2 – repeatability testing)				
	Summary statistics			Summary statistics			Difference from baseline		Summary statistics			Difference from Day 1	
Outcome	Median	IQR	Range	Median	IQR	Range	*p*-value	95% CI	Median	IQR	Range	*p*-value	95% CI
Two minute walk distance (2MWD), meters	135	130–163	17–233	141	127–163	14–239	0.984	−3.31-5	145	129–168	18–236	0.082	−2.47-4.82
Ten meter walk/run (10MWR), seconds	7.1	6.3–7.6	2.8–40.4	6.8	6.3–8	2.9–74	0.629	−0.2-0.29	7.3	6.5–8	2.8–58	0.269	−0.1–0
Four-stair climb (4SC), seconds	4.9	3.9–6.6	1.7–49.8	5.1	4.2–7.2	1.9–54.2	0.016	0.1–0.7	5.8	4.5–8.2	1.9–61	0.475	−0.35-0.58
Timed up-and-go (TUG), seconds	10	9–12	6–80	11	9–12	7–81	0.103	0–1	11	9–13	6–90	0.716	−0.82-0.82
Performance of upper limb (PUL)	42	37–42	30–42	42	37–42	29–42	0.553	−1-0	41.5	35–42	29–42	0.567	0–0
Manual muscle sum score (MMT)	182	167–198	145–262	186	175–200	142–258	0.158	−3-13					
Forced vital capacity (FVC), liters	2.905	2.45–3.71	1.98–5.94	2.97	2.53–3.57	1.96–5.76	0.647	−0.13-0.12					
Forced expiratory volume (FEV1), liters	2.535	2.26–2.97	1.53–4.72	2.56	1.88–2.94	1.55–4.69	0.053	−0.17-.004					
Mean inspiratory pressure (MIP), cmH2O	48.5	39–62	26–124	55	43–72	23–117	0.037	0.02–7.98					
Mean expiratory pressure (MEP), cmH2O	59.5	49–80	31–154	70	52–90	29–128	0.129	−3.96-13.97					
DEXA - lean body mass, kg	35.3	29.2–38.9	22.7–50.5	34.7	29.8–39	22.1–51.4	0.856	−0.46-0.73					
DEXA - body fat, kg	29.7	27.9–38.2	15.7–45.8	29.2	28.2–37	16.3–49.2	0.809	−1.03-0.83					
MRI - intramuscular fat fraction, %	67.9	57.8–72.9	36.2–90.3	69.3	62.4–72.8	38.1–88.5	0.266	−.072–1.86

#### Follow-up

Shapiro-Wilk testing showed that the normality assumption was not met for the 2MWD, 10MWR, TUG, 4SC, PUL, and therefore non-parametric tests were used to compare baseline to follow-up measurements. Because four participants did not complete the second day of repeatability testing, only values for the first day of repeatability testing were used in this analysis. Wilcoxon signed rank testing did not identify a statistically significant change in the 2MWD, 10MWR, TUG, or PUL. With the 4SC, there was an increase of 0.75 s between the baseline and 4-month visits with a *p*-value of 0.016 (Table 2).

Additionally, we compared baseline measurements of the MMT sum score, QMT (for each muscle group), FVC, FEV1, MIP, MEP, lean body mass, and total body fat from DEXA to the measurements at the 4-month follow-up visit. No statistically significant differences were detected in any of these outcomes over the follow-up period (Table 2). Performance on timed function testing did not vary significantly based on age, gender, body mass index, CK, or disease duration. With respect to genotype, the majority of participants were homozygous for the most common pathogenic variant of FKRP (NM_024301.5(FKRP): c.826C > A (p.Leu276Ile)). While LGMD2I patients who do not have this common variant reportedly have more severe phenotypes, there were too few subjects who were not homozygous for this pathogenic variant to assess phenotypic differences between different genotypes [3].

### Analysis of MRI outcomes

Shapiro-Wilk testing showed that the normality assumption was met for MRI muscle and fat fractions. Student’s t-tests did not show a statistically significant change in the muscle or fat fractions over the 4-month observation period. As the intramuscular fat fractions did not vary significantly over time, associations between the change in fat fraction and differences in clinical outcome measures could not be evaluated.

**Fig. 3:**
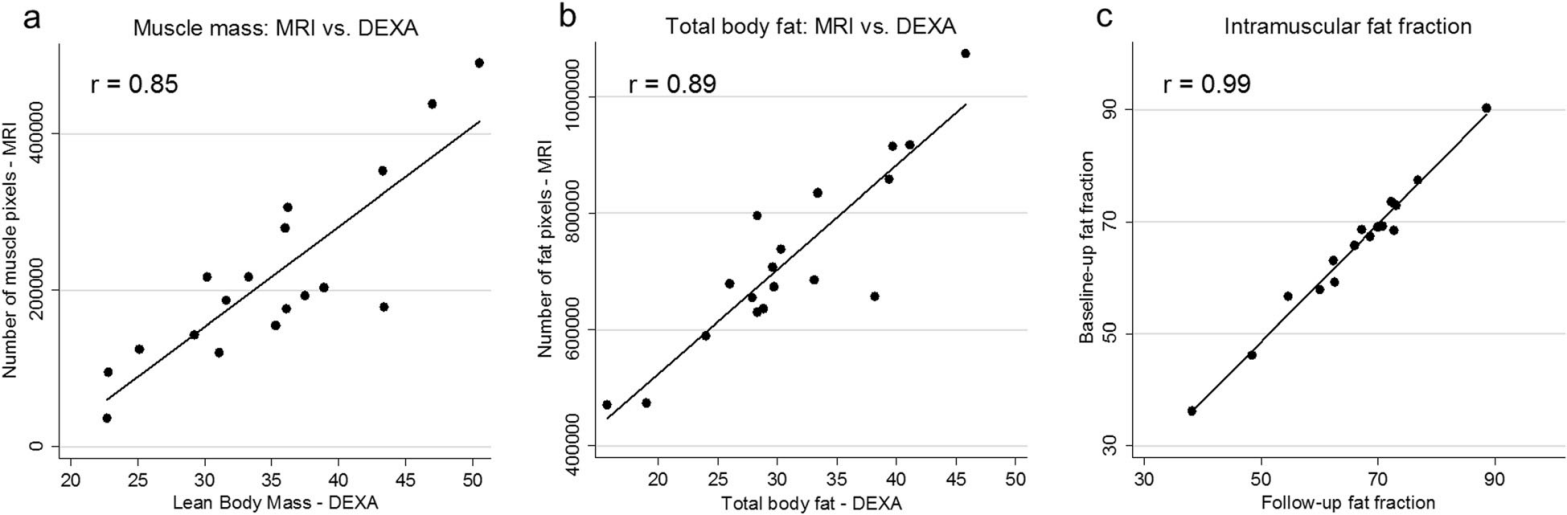
Body composition measures on MRI and DEXA scanning. The number of voxels identified as muscle strongly correlate with estimates of lean body mass on DEXA (3A). The number of voxels corresponding to body fat on MRI strongly correlate to DEXA estimates of total body fat (3B). Estimates of the intramuscular fat fraction are strongly correlated when comparing baseline to follow-up measurements

Estimates of lean body mass and body fat were obtained using DEXA scanning, and these values were compared to the number of voxels assigned to muscle or total body fat derived from MRI scanning. (Fig. 3).

**Fig. 4:**
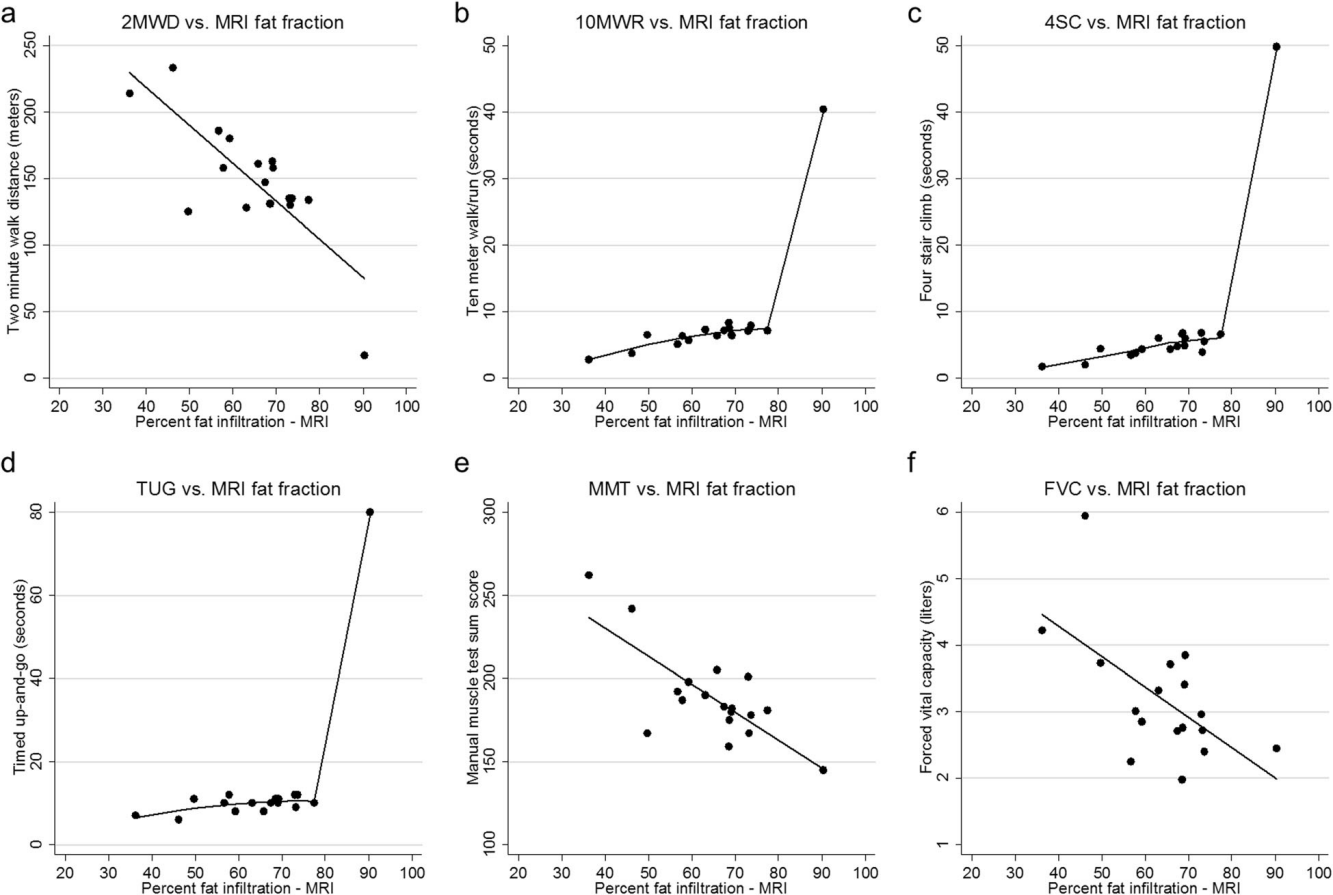
Timed function testing (2MWD, 10MWR, 4SC, TUG), manual muscle testing, and forced vital capacity as a function of the intramuscular fat fraction derived from MRI

We explored the associations between baseline fat fractions with the clinical outcome measures collected at the same visit through correlation analyses (Table 3). There were statistically significant linear associations between the MRI fat fractions with the 2MWT, PUL, and FVC. In the case of the 10MWR and 4SC, there appeared to be a linear association between the imaging and timed function measures for most of the data points. As data was sparse at the more severe disease range, it is unclear if the extreme values were true outliers or if performance times rise exponentially as the fat fraction approaches 100% (Fig. 4). The TUG was only weakly correlated to the thigh fat fraction in this study.

**Table 3:** Correlation between outcome measures taken at baseline

Outcome	DEXA – body fat	DEXA – LBM	MEP	MIP	FEV1	FVC	MMT	PUL	TUG	4SC	10MWR	2MWD
MRI muscle fat fraction (MFF), %	−0.027	−0.718	−0.535	− 0.448	− 0.572	− 0.605	− 0.759	− 0.267	0.565	0.700	0.733	− 0.794
	0.9149	0.0008	0.0268	0.0711	0.0164	0.01	0.0003*	0.2834	0.0227	0.0026	0.0013	0.0001*
Two minute walk distance (2MWD), meters	0.071	0.765	0.587	0.481	0.514	0.608	0.838	0.316	−0.555	−0.671	−0.846	NA
	0.7721	0.0001*	0.0105	0.0434	0.0290	0.0074	< 0.00001*	0.1873	0.0258	0.0044	0.0001*	
Ten meter walk/run (10MWR), seconds	−0.373	−0.855	− 0.641	− 0.465	−0.582	− 0.666	−0.783	− 0.103	0.549	0.805	NA	
	0.1546	< 0.00001*	0.0074	0.0693	0.0180	0.0048	0.0003*	0.7031	0.0276	0.0002*		
Four-stair climb (4SC), seconds	−0.138	−0.655	−0.356	−0.077	− 0.409	−0.402	− 0.560	−0.100	0.680	NA		
	0.6091	0.0059	0.1758	0.7780	0.1156	0.1229	0.0242	0.7129	0.0038			
Timed up-and-go (TUG), seconds	0.248	−0.493	−0.145	−0.287	−0.536	− 0.469	−0.549	− 0.342	NA			
	0.3544	0.0526	0.5915	0.2813	0.0323	0.0671	0.0276	0.1950				
Performance of upper limb (PUL)	−0.296	−0.082	0.054	0.134	0.327	0.303	0.388	NA				
	0.2186	0.7391	0.8302	0.5959	0.1854	0.2219	0.1003					
Manual muscle sum score (MMT)	0.205	0.792	0.816	0.757	0.665	0.721	NA					
	0.3997	0.0001*	< 0.00001*	0.0003*	0.0026	0.0007						
Forced vital capacity (FVC), liters	0.017	0.667	0.625	0.696	0.956	NA						
	0.946	0.0025	0.0073	0.0019	< 0.00001*							
Forced expiratory volume (FEV1), liters	−0.035	0.579	0.645	0.767	NA							
	0.8917	0.0119	0.0052	0.0003*								
Mean inspiratory pressure (MIP), cmH2O	0.162	0.642	0.928	NA								
	0.5210	0.0041	< 0.00001*									
Mean expiratory pressure (MEP), cmH2O	0.289	0.748	NA									
	0.2456	0.0004*										
DEXA - lean body mass (LBM), kg	0.374	NA										
	0.1143											
DEXA - body fat, kg	NA											

Pearson correlation coefficients (top) and *p*-values (bottom) are shown for the 2MWD, MMT, PFT, DEXA, and MRI data. Spearman correlation coefficients are shown for the FSC, TUG, and 10MWR due to non-normal distribution. Asterisks note statistically significant *p*-values under a Bonferroni adjustment

A sensitivity analysis was performed excluding the most extreme outlying data point. The exclusion of this data point increased the linear correlation between the fat fraction and several timed function tests; the r value increased from 0.605 to 0.803 for the 4SC and from 0.631 to 0.853 for the 10MWR. The exclusion of outliers did not greatly improve the linear correlation of the TUG (r = 0.559 vs. 0.561).

## Discussion

In this study, we examined various clinical outcomes measures that are commonly used in muscular dystrophy trials. The findings of this study are largely consistent with prior studies in LGMD2I, which did not detect significant changes in muscle strength and function over a 12-month period but did find gradual progression of weakness over years [26, 27]. These studies in LGMD2I were able to demonstrate measurable changes in fat infiltration within individual muscles over 12 months. In our study, we did not detect significant changes in thigh fat fractions. This is likely due to the short period of follow-up, although it is also possible that small but measurable changes within individual muscles would not significantly impact the total muscle and fat volumes.

The results of our study differ from previous studies with respect to a single measure, the 4SC, in which a statistically significant difference was observed over 4 months. There are several possible reasons for this contradictory finding. The small sample size of this trial allows extreme values in a few participants to have a large statistical impact, and the statistical significance of the observed difference was largely driven by two outliers whose 4SC times worsened by 3.4 and 4 s between the baseline and follow-up visits. While this could represent a more rapid progression of disease in the more severely affected participants, the performance of these participants also varied more widely on repeatability testing than the rest of the study sample (Fig. 2). Similar variability in the 10MWR and the TUG may indicate that the shorter timed function tests that require a brief burst of physical exertion may be more susceptible to confounding by day-to-day variations in effort and fatigue among the more severely affected participants. Exclusion of the outliers in our sensitivity analysis strengthened the linear correlations between the MRI fat fraction and function tests like the 4SC and 10MWR, but also weakened the significance of the change in the 4SC. In designing clinical trials in this disease population, investigators may need to use analysis strategies that can compensate for unequal variances. This may include stratifying enrollment based on disease severity, adjusting for baseline performance when performing statistical analyses, or limiting enrollment to a specific disease subgroup or a specific range of performance on timed function tests.

Conversely, it is important to note the outcomes that show more consistent variability across all levels of disease severity. In our repeatability analysis, the 2MWD and PUL showed relatively uniform variability across the full cohort, suggesting that they may be robust measures in terms of day-to-day variability across a wider range of disease severity in LGMD2I. Among the outcome measures that were not tested for repeatability, the MMT sum score, pulmonary function tests, and DEXA measurements showed consistent variability when comparing baseline and follow-up measurements.

The methodology used to perform the MRI analysis offers several important advantages in studying the muscular dystrophy population. Although radiologists inspected the segmented images for processing and segmentation errors, the segmentation of tissues was entirely automated. By doing so, there is a reduced risk of ascertainment bias compared to protocols in which interpreters score each muscle or define the muscle borders manually. Another important advancement in this technique is the ability to include all slices acquired in the thigh (50–60 slices per subject). Prior studies that have employed manual segmentation of tissues have found the process to be prohibitively time-consuming, and many of them limit the analysis to a few slices. While this may be appropriate for some studies, not using all of the acquired data represents a significant compromise to what is one of the major advantages of scanning, which is the ability to examine large regions of the body in diseases that affect the whole body. A third significant advancement is the ability to distinguish intramuscular fat replacement from extramuscular subcutaneous fat through unique radiographic tissue signatures.

Our analysis did not show a statistically significant change in thigh fat fractions over the 4-month observation period. This is a relatively short period of follow-up, and it is therefore possible that no significant change in whole-body fat fraction has occurred (although small changes within individual muscles may not be detected using this method). Under this assumption, the test-retest reliability of the fat fractions over the follow-up period appears to be excellent, with similar variability across the spectrum of disease severity. Our preliminary analysis also suggests that the segmentation method can reliably quantify body fat and muscle (compared to DEXA measures of body composition) and that MRI fat fractions show a strong linear correlation to the 2MWD and the MMT sum score across all levels of disease severity.

There are several limitations to this study, the most significant of which is the sample size. The sample size is comparable to other phase 1b/2a trials in neuromuscular diseases but is likely to be underpowered to detect small changes in the outcome measures that were collected. The small size of the study also makes it more vulnerable to the influence of outliers, which are found in our sample. Our decision to include the extreme outliers in our analysis limits the confidence with which we can ascribe changes in outcomes to true disease progression, such as with the 4SC. However, the exclusion of properly obtained data would risk underestimating the variability in the study population and overestimating the power to detect change in our outcome measures. The duration of follow-up was also fairly short and likely did not allow sufficient time for measurable pathologic changes to occur in the outcomes studied. We therefore cannot comment on the performance of these biomarkers with respect to sensitivity to change. Additional limitations relate to the tissue segmentation technique. While the deep learning models can automatically segment tissue types, they are currently unable to resolve individual muscles or muscle groups. Therefore, different rates of disease progression between muscles and patterns of preferential muscle involvement or sparing cannot be assessed. We have also observed that while tissue signatures of extensively fat infiltrated muscle are distinguishable from subcutaneous fat, large confluent regions of completely fat infiltrated muscle can have a tissue signatures that are difficult to distinguish from subcutaneous fat. Therefore, there is a greater chance of misclassifying the tissue signatures among subjects with almost complete fat replacement in extensive regions of muscle. We believe that this risk is mitigated by our eligibility criteria, which requires that participants independently stand and walk (which generally requires some intact muscle in the regions being analyzed). However, future studies may need to account for this limitation in the eligibility or analysis phases.

## Conclusions

The clinical trial lead-in data examined in this analysis offer valuable insight into the strengths and limitations of commonly used outcome measures in neuromuscular diseases. We also introduce a novel method that uses deep learning techniques to produce a fully automated analysis of whole-body MRI scans. These techniques allow for the calculation of intramuscular fat and muscle fractions that show strong repeatability in a diverse study sample. Future studies of this technique will establish the ability of these novel techniques to detect changes in the volume of intramuscular fat and muscle.

## Data Availability

Data used and/or analyzed during the current study are also available from the principal investigator (Dr. Kathryn Wagner) on reasonable request.

## Abbreviations

2MWD: Two-minute walk distance
4SC: Four-stair climb
10MWR: 10-m walk/run
CK: Creatine kinase
CNN: Convolutional neural networks
CTM: Continuous table movement
DEXA: Dual-energy X-ray absorptiometry
FDA: Food and Drug Administration
FEV1: Forced expiratory volume
FKRP: Fukutin-related protein
FVC: Forced vital capacity
LGMD2I: Limb-girdle muscular dystrophy, type 2I
MEP: Mean expiratory pressure
MIP: Mean inspiratory pressure
MMT: Manual muscle testing
MPDL: Multiparametric deep learning
MRC: Medical Research Council
MRI: Magnetic resonance imaging
PUL: Performance of Upper Limb assessment
QMT: Quantitative muscle testing
STIR: Short tau inversion recovery
TS: Tissue signature
TUG: Timed up and go
WBMRI: Whole-body magnetic resonance imaging
